# Public engagement in outcomes development - three degrees of separation

**DOI:** 10.1186/1745-6215-16-S3-O3

**Published:** 2015-11-24

**Authors:** Sally Crowe, Sandra Regan, Ann Daly

**Affiliations:** 1 Crowe Associates, Thame Oxon, OX9 3LW, UK

## Background

This presentation will focus on a UK Cochrane funded project that explored different ways of engaging patients, the public and health practitioners in the development of outcomes for systematic reviews. It is called 'Outcomes Most Important for Patients, Public and Practitioners (OMIPPP)'.

## Method

Working with three Cochrane Review Groups (CRG); Airways, Ear Nose and Throat (ENT) and Pregnancy and Childbirth we focussed on outcomes for reviews in Asthma, Rhinosinusitis and Breastfeeding respectively. For each group we used a different method to engage; for asthma we facilitated a full day workshop. Working in partnership with Asthma UK, we prepared for this by gathering perspectives of asthma via a Facebook survey, and reviewing existing core outcome sets. Working in partnership with evidENT we gathered perspectives in rhinosinusitis using an online survey and experimented with social media as a way of reaching out beyond their networks as there are no relevant patient groups. We compared survey findings with existing outcomes used for reviews of chronic sinusitis. For breastfeeding we worked with the National Childbirth Trust and the Breastfeeding Network to review an existing online collection of experiences of breastfeeding called Healthtalk (http://www.healthtalk.org). Healthtalk researchers reanalysed the original data for clues to outcomes. These were shared, discussed and compared with existing outcomes used in systematic reviews of breastfeeding interventions.

## Results

At the time of writing this abstract the project is not yet complete, however early results will be discussed. We are interested in the following aspects of evaluation; how relevant was the gathered data for systematic review outcomes? What were the cost and resource implications of each method? From a review group perspective how feasible are these methods? Has the project extended the reach of CRGs with interested public/patient and practitioner groups? How does our data compare with other outcomes exercises e.g. COMET?

